# Supplementary material for: Travel fosters tool use in wild chimpanzees
Source: eLife. 2016 Jul 19;5:e16371. doi: 10.7554/eLife.16371 (PMC4972540; doi:10.7554/eLife.16371)
Supplement: Source code 1. — DOI: http://dx.doi.org/10.7554/eLife.16371.016 [file elife-16371-code1.pdf]

datasets and model specifications for 'Travel fosters tool use in wild chimpanzees'

Thibaud Gruber, Klaus Zuberbühler and Christof Neumann

Engagement model

Data are in "engagementdata.csv". Columns contain the following information:

- **experimentID**: categorical, experiment ID
- **ID**: categorical, subject ID
- **engaged**: numeric, time engaged with experiment
- **ripefruit**: numeric, proportion of ripe fruit feeding
- **timeframe**: numeric, time period prior to trial considered considered
- **travelprop**: numeric, proportion of time spent travelling
- **sex**: categorical, sex of subject
- **age**: numeric, age of subject
- **tooluse**: categorical, did the subject use a tool or not
- **autocor**: numeric, auto-correlation term

The full and null models were specified in the following way:

```
library(lme4)
xdata <- read.csv("engagementdata.csv")

res <- lmer(engaged ~ ripefruit *timeframe *travelprop +sex +age +tooluse +autocor
            + (1 +ripefruit +travelprop |ID) + (1 +tooluse |experimentID),
            xdata, REML=F)

null <- lmer(engaged ~ autocor
             + (1 +ripefruit +travelprop |ID) + (1 +tooluse |experimentID),
             xdata, REML=F)

# full model versus null
anova(null, res)
```

Table 1 in the manuscript was obtained with the following commands:

```
coefficients(summary(res))
confint(res, method="Wald")
drop1(res, test="Chisq")
```

Tool use model

Data are in three files "tooldata1.csv", "tooldata7.csv" and "tooldata13.csv", which correspond to the three time periods we tested separately. Columns contain the following information:

- **experimentID**: categorical, experiment ID

- **ID**: categorical, subject ID
- **tooluse**: numeric, did the subject use a tool (=1) or not (=0)
- **ripefruit**: numeric, proportion of ripe fruit feeding
- **travelprop**: numeric, proportion of time spent travelling
- **sex**: categorical, sex of subject
- **age**: numeric, age of subject
- **engaged**: numeric, time engaged with experiment
- **autocor**: numeric, auto-correlation term

Models were constructed in the following way:

```
# time period: 1 week
xdata <- read.csv("tooldata1.csv")

# full model
res <- glmer(tooluse ~ ripefruit*travelprop +sex +age +autocor
             +(1 |ID) +(1 |experimentID), xdata, family=binomial, offset=engaged,
             glmerControl(optimizer="bobyqa", optCtrl = list(maxfun = 1000000)))

null <- glmer(tooluse ~ autocor
              +(1 |ID) +(1 |experimentID), xdata, family=binomial, offset=engaged,
              glmerControl(optimizer="bobyqa", optCtrl = list(maxfun = 1000000)))
# full model versus null
anova(null, res) # Table 2

# remove interaction
red <- glmer(tooluse ~ ripefruit +travelprop +sex +age +autocor
             +(1 |ID) +(1 |experimentID), xdata, family=binomial, offset=engaged,
             glmerControl(optimizer="bobyqa", optCtrl = list(maxfun = 1000000)))
anova(red, res) # discard interaction term (Table 2)

# Table 3:
coefficients(summary(red))
```

Community comparison

Data are in “crosscommunity.csv”. Columns contain the following information (details in Table 4):

- **tools**: numeric, size of tool repertoire
- **dailypath**: numeric, daily travel path length (km)
- **pct.fruit**: numeric, percentage of fruit in diet
- **pct.travel**: numeric, percentage of travel in activity budget

```
xdata <- read.csv("crosscommunity.csv")

plot(0,0, xlim=c(0,100), ylim=c(0,15), "n", xlab="Percent fruit",
     ylab="Size of the tool repertoire", las=1)
points(xdata$pct.fruit, xdata$tools)
cor(xdata$pct.fruit, xdata$tools, method="s", use="c")

plot(0,0, xlim=c(0,25), ylim=c(0,15), "n", xlab="Percent travel",
```

```r
     ylab="Size of the tool repertoire", las=1)
points(xdata$pct.travel, xdata$tools)
cor(xdata$pct.travel, xdata$tools, method="s", use="c")

plot(0,0, xlim=c(0,5), ylim=c(0,15), "n", xlab="Daily travel distance (km)",
     ylab="Size of the tool repertoire", las=1)
points(xdata$dailypath, xdata$tools)
cor(xdata$dailypath, xdata$tools, method="s", use="c")
```
